# Utility of Video Laryngoscopy in Diagnosing Unanticipated Gastro-Tracheal Fistula Following Intubation for Supratentorial Craniotomy

**DOI:** 10.7759/cureus.69499

**Published:** 2024-09-16

**Authors:** Syed Muhammad Hussain Rizvi, Muhammad Irfan Ul Haq, Faraz Shafiq

**Affiliations:** 1 Anesthesiology, Aga Khan University, Karachi, PAK; 2 Anesthesiology, North Cumbria Integrated Care NHS Foundation Trust, Cumbria, GBR

**Keywords:** craniotomy, esophagectomy, fistula, intubation, laryngoscopes

## Abstract

Gastro-tracheal fistula (GTF) is a rare but serious complication that can occur after esophagectomy. It involves an abnormal connection between the gastric conduit and the trachea that allows gastric contents to enter the respiratory tract. Although GTF is uncommon, it can lead to severe complications such as aspiration pneumonia, respiratory distress, mediastinitis, and sepsis. This case report details the management of an unexpected air leak in a 55-year-old female with a history of esophagectomy who was scheduled for an urgent supratentorial craniotomy. During anesthesia induction, a significant air leak was detected using a McGrath video laryngoscopy (VDL). This tool helped identify the leak and led to further investigation with fiberoptic bronchoscopy, which uncovered a GTF originating from the right main bronchus. The surgical procedure was postponed for a multidisciplinary assessment, and esophageal stenting was later used to manage the fistula successfully. This case highlights the crucial role of VDL in the early detection and management of complex airway issues, demonstrating its importance in enhancing patient outcomes through improved decision-making and interdisciplinary collaboration.

## Introduction

Gastro-tracheal fistula (GTF) is a rare but potentially life-threatening complication of esophagectomy. It is characterized by an abnormal connection between the gastric conduit and the trachea, allowing gastric contents to enter the respiratory tract. Although GTF is infrequently encountered, with only a limited number of cases documented in the medical literature [1], it presents significant risks due to potential complications such as aspiration pneumonia, respiratory distress, mediastinitis, and sepsis [2].

Clinically, GTF can manifest with a combination of respiratory and gastrointestinal symptoms. Patients may experience chronic cough, dyspnea, and hemoptysis, as well as dysphagia, regurgitation, and unexplained weight loss. Airway management in such cases becomes particularly challenging, especially in emergency situations requiring urgent surgical intervention. A persistent air leak detected during anesthesia induction or ventilation should raise suspicion of GTF in at-risk patients.

This case report underscores the management of unexpected air leaks in elderly patients with a history of esophagectomy. The use of video laryngoscopy (VDL) was instrumental not only in the early detection of leaks but also in facilitating timely decision-making and intervention.

## Case presentation

The reported patient was a 55-year-old female, a retired school teacher residing in a rural area with limited healthcare facilities. Living alone and receiving care from her students for chronic health issues, she had undergone neoadjuvant concurrent chemoradiation therapy followed by a two-stage esophagectomy for esophageal carcinoma several years prior. Post-procedure, she developed a chronic cough and dyspnea, for which a workup was advised, but she missed her subsequent follow-ups.

**Figure 1:**
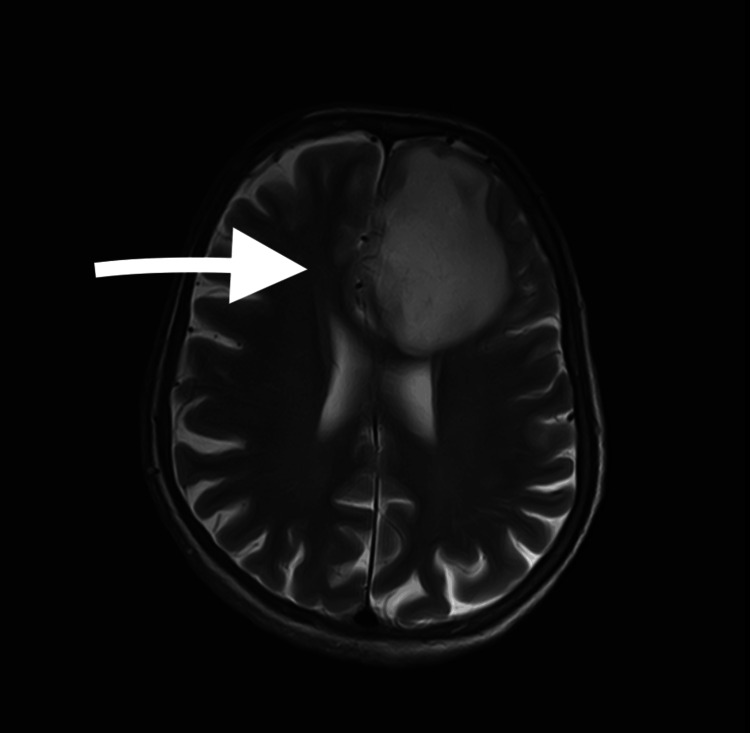
MRI image demonstrating the tumor The arrow highlights a hemorrhagic metastatic deposit in the left frontal lobe, accompanied by surrounding vasogenic edema that is causing a mass effect and a midline shift.

**Figure 2:**
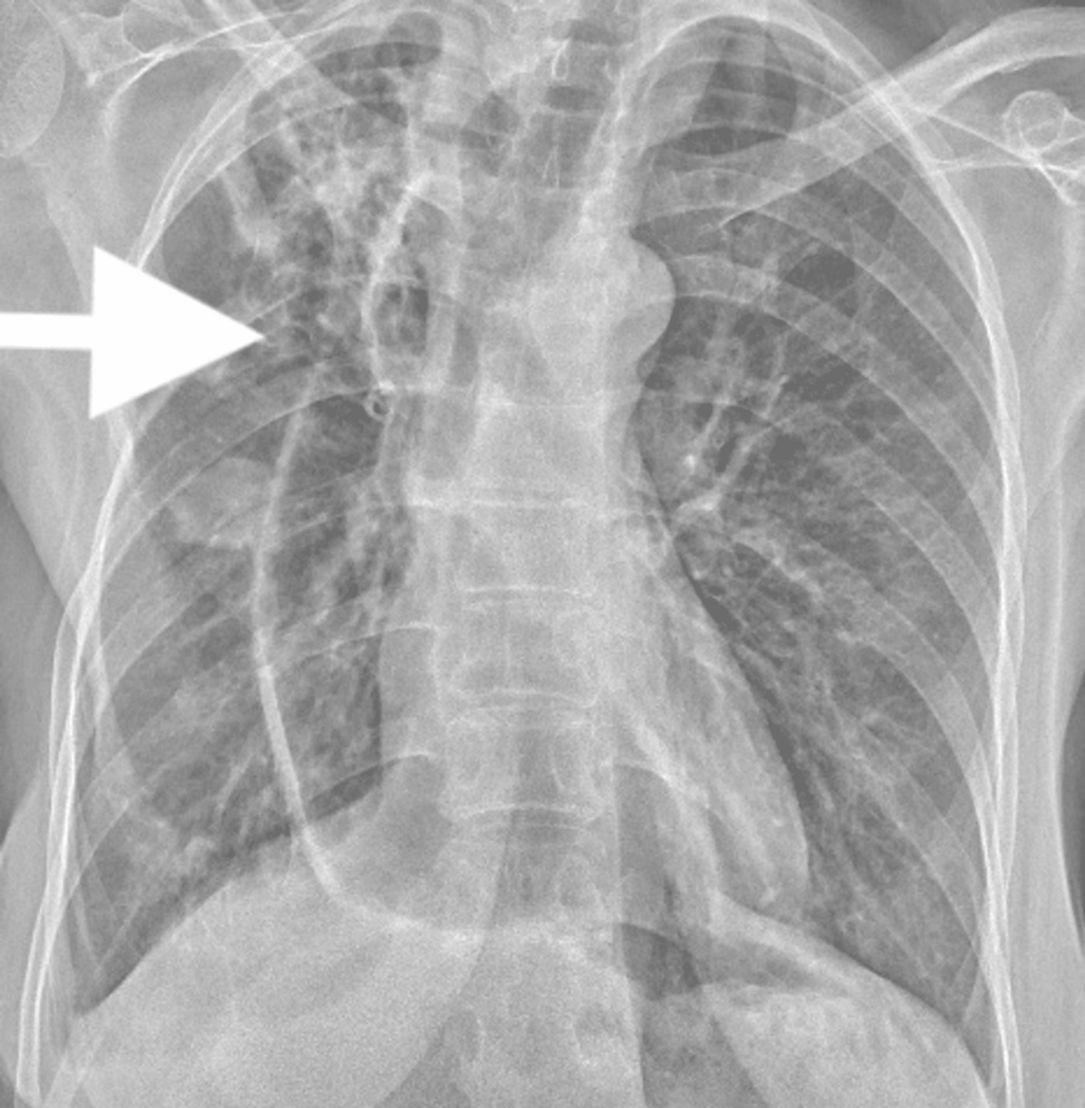
Chest X-ray The arrow indicates a large, lobulated nodularity in the right mid-zone, which is likely indicative of an infection or metastases.

Recently, she presented to the hospital with drowsiness, confusion for one day, and a chronic cough that had persisted for two months. Imaging studies (Figure 1) revealed an intracranial hemorrhagic metastatic deposit in the left frontal lobe. She was scheduled for an urgent supratentorial craniotomy and resection of the lesion. Given her symptoms and preoperative chest X-ray findings (Figure 2), a pulmonology consultation was requested. The pulmonologist suggested that the changes might be related to either a chronic infection or metastases.

 Table 1 presents the laboratory investigations.

**Table 1:** Lab investigations

Parameters	Results	Reference range
HB	11.4 gm/dl	12.3-16.6
HCT	39.9	38.4-50.7
WBC	26.6 × 10E9/L	4.8-11.3
PLT	364 × 10E9/L	154-433
Parameters	Results	Reference range
Na	140 mmol/L	136-145
K	3.8 mmol/L	3.5-5.1
Cl	104 mmol/L	98-107
HCO3	24.2 mmol/L	22-29
Cr	0.5 mg/dL	0.5-1
Parameters	Results	Reference range
PT	10.3 seconds	9.3-12.8 seconds
INR	1.0	0.9-1.2

General anesthesia with controlled ventilation and intubation was planned. Routine ASA standard monitoring was applied. Following a straightforward induction, bag-mask ventilation, and intubation using a McGrath VDL, a significant air leak was noted, which hindered the achievement of the desired tidal volumes. A subsequent laryngoscopy revealed a pronounced leak from the glottic area, despite adequate cuff pressure and correct endotracheal tube positioning.

**Figure 3:**
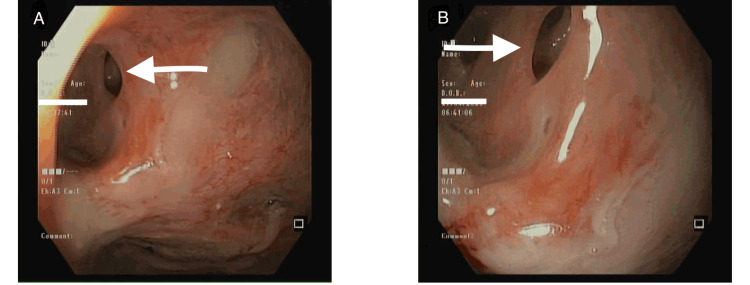
Endoscopic image demonstrating the presence of a gastrotracheal fistula Arrows in A and B highlight a large fistulous opening in the esophagus.

Based on the VDL findings, a fiberoptic bronchoscopy (FOB) was planned to confirm the source of the leak. The FOB revealed an abnormal opening in the right main bronchus, leading to a provisional diagnosis of GTF. Due to the complexity of the case, a thorough discussion was held among the surgical team, anesthesia team, and caregivers, weighing the risks and benefits. The surgery was postponed, and the patient was reversed from anesthesia. She remained stable and was transferred back to the ward with instructions to follow up with gastroenterology. Subsequent endoscopy (Figure 3) confirmed the presence of a fistula.

## Discussion

GTF is an extremely rare complication following esophagectomy, with an incidence reported between 0.3% and 1.5% [3]. It involves an abnormal connection between the airway and the reconstructed stomach, which has been repositioned during esophageal resection. This fistulous path allows gastric secretions to spill into the tracheobronchial tree, leading to typical symptoms such as cough and chronic respiratory tract infections. Although GTF is more common in patients who have undergone radiation therapy, anastomotic leaks from the gastric conduit are the most frequent cause of this rare complication [4].

GTF is associated with significant morbidity, including respiratory failure, lung infections, and a high mortality rate of approximately 57%, primarily due to septic shock [5,6]. Diagnosing GTF is challenging due to its low incidence and overlapping symptoms. Effective anesthetic management requires a thorough preoperative assessment, including evaluation of the fistulous opening’s anatomical location, size, and symptom severity.

VDL is valuable not only for intubation but also in managing challenging airway scenarios where prompt decision-making is crucial [7,8]. VDL enhances situational awareness and improves communication with the surgical team [9]. In our case, despite the urgency of the surgery, the decision to postpone was made easier by the VDL findings.

Typically, patients with GTF require one-lung ventilation (OLV) as part of their anesthetic management [10]. We considered OLV for this case but decided against it due to the high volume of the leak, the patient’s baseline lung condition, and the nature of the craniotomy procedure. Airway leaks can lead to alveolar hypoventilation, desaturation, hypercapnia, and aspiration of gastric contents [11]. Effective management of such complex airway scenarios requires both technical and nontechnical skills, as human error tends to increase with difficulty and failed attempts [12].

In this case, VDL was instrumental in identifying the leak and guiding subsequent diagnosis with FOB. A CT scan revealed abnormal communication between the gastric conduit and the right upper lobe at the T5 vertebral level. A multidisciplinary approach, including consultation with gastroenterology, was undertaken. The patient underwent esophageal stenting under monitored anesthesia care, with the successful placement of a covered metallic stent at the site of the fistula. The patient remained hemodynamically stable and was discharged home after two days.

## Conclusions

This case report underscores the valuable role of VDL in diagnosing unexpected GTF during the induction of anesthesia for an urgent supratentorial craniotomy. Early recognition and prompt intervention are crucial for achieving the best possible outcomes in challenging airway scenarios. VDL has demonstrated significant safety benefits by providing both technical and nontechnical support and facilitating shared decision-making.

Our findings emphasize the importance of advanced airway management tools in improving situational awareness and supporting critical decision-making in complex clinical situations. The use of VDL not only enabled accurate diagnosis but also enhanced patient safety by mitigating potential complications related to GTF during an urgent surgical procedure. This case highlights the necessity of a collaborative, multidisciplinary approach in managing rare and potentially life-threatening complications, ultimately leading to improved patient outcomes.
